# 1α,25-Dihydroxyvitamin D_3_ Improves Follicular Development and Steroid Hormone Biosynthesis by Regulating Vitamin D Receptor in the Layers Model

**DOI:** 10.3390/cimb45050256

**Published:** 2023-05-04

**Authors:** Manman Cheng, Zhenquan Song, Yan Guo, Xuliang Luo, Xuelian Li, Xiaohui Wu, Yanzhang Gong

**Affiliations:** Key Laboratory of Agricultural Animal Genetics, Breeding and Reproduction of Ministry of Education, College of Animal Science and Technology, Huazhong Agricultural University, Wuhan 430070, China

**Keywords:** young layer, 1α, 25-dihydroxy vitamin D_3_, vitamin D receptor, granulosa cells, theca cells, steroid hormones

## Abstract

1α,25-Dihydroxyvitamin D_3_ (VitD_3_) is the active form of vitamin D, and it regulates gene expression and protein synthesis in mammalian follicle development. However, the function of VitD_3_ in the follicular development of layers remains unclear. This study investigated, through in vivo and in vitro experiments, the effects of VitD_3_ on follicle development and steroid hormone biosynthesis in young layers. In vivo, ninety 18-week-old Hy-Line Brown laying hens were randomly divided into three groups for different treatments of VitD_3_ (0, 10, and 100 μg/kg). VitD_3_ supplementation promoted follicle development, increasing the number of small yellow follicles (SYFs) and large yellow follicles (LYFs) and the thickness of the granulosa layer (GL) of SYFs. Transcriptome analysis revealed that VitD_3_ supplementation altered gene expression in the ovarian steroidogenesis, cholesterol metabolism, and glycerolipid metabolism signaling pathways. Steroid hormone-targeted metabolomics profiling identified 20 steroid hormones altered by VitD_3_ treatment, with 5 being significantly different among the groups. In vitro, it was found that VitD_3_ increased cell proliferation, promoted cell-cycle progression, regulated the expression of cell-cycle-related genes, and inhibited the apoptosis of granulosa cells from pre-hierarchical follicles (phGCs) and theca cells from prehierarchical follicles (phTCs). In addition, the steroid hormone biosynthesis-related genes, estradiol (E2) and progesterone (P4) concentrations, and vitamin D receptor (VDR) expression level was significantly altered by VitD_3_. Our findings identified that VitD_3_ altered the gene expression related to steroid metabolism and the production of testosterone, estradiol, and progesterone in the pre-hierarchical follicles (PHFs), resulting in positive effects on poultry follicular development.

## 1. Introduction

Vitamin D is an essential nutrient for the regulation of several physiological activities, involved in calcium and phosphate homeostasis and skeleton calcification, with vitamin D deficiency linked to decreased egg production and eggshell quality deterioration in poultry [1,2]. Animals can obtain 1α,25-dihydroxyvitamin D_3_ (VitD_3_) through dietary intake or through synthesis in the skin, with 25-hydroxylation in the liver forming 25-hydroxyvitamin D_3_ followed by 1α-hydroxylation in the kidneys to form the active form of VitD_3_ [3]. The effect of VitD_3_ on shell quality and bone strength has been reported in laying hens. Bar et al. [4] found that VitD_3_ supplementation in thin-shelled hens significantly increased eggshell thickness. In addition, VitD_3_ supplementation could increase the tibia-breaking strength in aged hens [5]. Recently, the role of VitD_3_ in regulating follicular development has been gradually verified, and it was found that VitD_3_ increased follicle survival and growth in in vitro cultured follicles [6,7].

Studies in mammals have demonstrated that VitD_3_ is indispensable for ovarian function [8]. Vitamin D deficiency in females was related to arrested follicular development and prolonged estrous cycles, with fewer oocytes, ovulation disorders, and even infertility [9]. Studies in polycystic ovary syndrome (PCOS) patients showed that VitD_3_ supplementation promoted follicle growth, dominant follicle formation, and increased pregnancy rate and maintained an average menstrual cycle [10]. In addition, in the macaque, VitD_3_ supplementation promoted oocyte growth and folliculogenesis in in vitro cultured follicles [7]. These findings suggested that VitD_3_ alters ovarian functions, and it might be mediated by endocrine and paracrine signaling mechanisms. The hen ovary serves as an important model for studying follicular development and recruitment in reproductive biology [11]. In this study, we chose young layers (18 weeks old) as our experimental animal, as their follicles are usually arranged in a strict follicular hierarchy, and the ovulation-related hormones are secreted irregularly during this time.

Although it was found that VitD_3_ regulated ovary function in mammals, whether VitD_3_ has effects on the chicken ovary and also the mechanisms of VitD_3_ in regulating follicle development are still unclear. Since there is a follicular ovulation hierarchy in hens, few follicles can be selected and develop to maturity, and most growing follicles undergo follicular atresia [12]. Follicle selection in hens is described as the process of selecting one follicle per day from a pool of small yellow follicles to enter the preovulatory hierarchy, and the follicle selection process has been widely considered to be the rate-limiting step in the reproductive performance of hens [13,14]. The follicular development and follicle selection determine the number of mature follicles, which are key factors affecting poultry reproductive performance, especially egg production rate. Hence, it is of significant importance to illustrate the molecular mechanisms of VitD_3_ on the regulation of follicular development and follicle selection in the hen ovary model. In this study, we explored the direct action of VitD_3_ on ovarian follicle development and steroid hormone biosynthesis in vitro and in vivo in young layers (18–22 weeks old). The intention of this research was to investigate the direct function that VitD_3_ exerts on the follicular development of young layers, as well as its molecular mechanism.

## 2. Materials and Methods

### 2.1. Experimental Animals, Housing, and Experimental Design

Ninety 18-week-old Hy-Line Brown laying hens, of similar body weight (BW, 1.49 ± 0.05 kg), were used. All of the layers were housed in a controlled environment with light intensity of 20 Lx (lights on from 06:00–20:00), and during the experimental period, these layers had free access to water and feed. The animal procedures were approved by the Animal Care and Use Committee of Huazhong Agricultural University (HZAUCH-2020-001; 10 June 2020). These young layers were randomly divided into three groups with 30 layers per group for different treatments of VitD_3_ (calcitriol): the control (CON, with 0 μg/kg BW), low dosage (LVD, with 10 μg/kg BW), and high dosage (HVD, with 100 μg/kg BW). Calcitriol (Med Chem Express, Monmouth, NJ, USA) was administered by oral gavage, five times a week for 4 weeks. Dimethyl-sulfoxide (DMSO, Sigma, St. Louis, MO, USA) in corn oil served as a vehicle. As for the cell culture, small yellow follicles (SYFs, 6–8 mm) were taken from at least four hens and were harvested 10 h after laying. The layers were sacrificed using direct cervical dislocation based on the approval of the Animal Care and Use Committee.

### 2.2. Tissue Collection

The abdominal cavity of each hen was opened, and the functional ovary was carefully removed. Blood was collected at the same time. The entire ovary was immersed in PBS several times, and then the follicles were classified by size. Counting of the follicles was carried out according to previous reports [15,16], including small yellow follicles (SYFs, 6–8 mm), large yellow follicles (LYFs, 9–12 mm), and the preovulatory follicles (POFs, F1–F6). Then, the SYFs were collected for histological, RNA-seq, targeted metabonomics, and molecular analyses.

### 2.3. Measurement of Metabolite Contents in Serum and Cell Culture Media

Blood samples (5 mL) were taken from the wing vein of hens using centrifugal tubes for 2 h at room temperature and then centrifuged at 3000× *g* for 15 min to discard the blood cells. The serum was stored at −80 °C. The concentrations of follicle-stimulating hormone (FSH), luteinizing hormone (LH), estradiol (E2), progesterone (P4), androstenedione (ASD), anti-Müllerian hormone (AMH), triglycerides (TGs), and total cholesterol (TCHO) in serum were measured by ELISA kits. In the cell experiments, the cultured medium was harvested after the treatment of phGCs and phTCs with VitD_3_ for 48 h, and E2 and P4 concentrations were measured by ELISA kits. These measurements all followed the ELISA kit manufacturer’s instructions (Shanghai FANKEL Industrial Co., Ltd., Shanghai, China). Absorption at 450 nm was measured by using an Enspire Microplate Reader (PerkinElmer, EnSpire, Singapore). The sample concentrations were calculated according to the mean absorbance.

### 2.4. RNA Isolation, RT-qPCR, and RNA-Seq

TRIzol Reagent (Invitrogen, Carlsbad, CA, USA) was used for total RNA extraction, and then the total RNA was reversed to cDNA by the cDNA Reverse Transcription Kit (Vazyme, Nanjing, China). Real-time quantitative polymerase chain reaction (RT-qPCR) was conducted using the SYBR Green PCR kit (Bio-Rad, Hercules, CA, USA) for PCR amplification conditions as follows: first, denaturing at 95 °C for 5 min, followed by 35 cycles at 95 °C for 30 s, 60 °C for 1 min, 72 °C for 30 s, and finally 72 °C for 10 min. We used the 2^−ΔΔCT^ method for the calculation of relative gene expression levels and selected the housekeeping gene *GAPDH* as the internal control. The quantitative primers according to the mRNA sequences on NCBI and the details of the primer sequences are shown in Supplementary Table S1.

As for RNA-seq, we prepared RNA-Seq libraries with three biological replicates on an Illumina HiSeq 2500 platform by Gene Denovo Biotchnology Co. (Guangzhou, China) according to the manufacturer’s protocol [17,18]. The library generation includes enriching eukaryotic mRNA using Oligo (dT), adding fragmentation buffer to break the mRNA. These random hexamers were used for the first cDNA strand synthesis. After that, DNA polymerase I, dNTPs, RNase H, and buffer were added to synthesize the second cDNA strand. The resulting double-stranded cDNA was purified using AMPure XP beads, followed by end-repair, A-tailing, and ligation of sequencing adapters. Fragment size selection was executed with AMPure XP beads, and PCR amplification was used to enrich the cDNA library. Subsequently, the Illumina HiSeq 2500 platform (Illumina, San Diego, CA, USA) was employed to sequence the libraries according to Illumina’s RNA-seq instructions, generating 150 bp paired-end reads, which were further analyzed.

The low-quality reads and adapter reads were removed by fastp software [19]. We removed reads that contained adapters, those with over 10% of unknown nucleotides (Ns), and low-quality reads with more than 50% of bases (Q-value ≤ 20). The high-quality reads were aligned to the chicken reference genome (GRCg6a) by HISAT2 [20], gene count was generated by HTSeq [21], and the FPKM approach was utilized to evaluate the expression levels of genes. Then, differentially expressed genes were detected by DESeq2 (1.16.1, Illumina, San Diego, CA, USA) [22], whose cutoff was *p*-value < 0.05 and fold change > 1.5. We used clusterProfile to detect significant pathways based on the Kyoto Encyclopedia of Genes and Genomes (KEGG) database [23], and the pathways with *p*-value < 0.05 were considered as the significant pathways.

### 2.5. Analysis of Steroidogenesis-Targeted Metabolomics

The steroid hormones in the SYF fluid were measured using UPLC-QQQ-MS. The samples were analyzed on an AB SCIEX 5500 QQQ-MS system (AB SCIEX, Framingham, MA, USA) equipped with a Waters Acquity UPLC (Waters Corporation, Milford, USA) device. Six biological replicates were carried out for targeted metabolomics. For the quantification of steroid hormones, 200 μL SYF fluid was mixed with 50 μL standards, and then 500 μL methanol (Sigma, USA) and 500 μL acetonitrile (Sigma, USA) were added. Following this, the samples were vortexed and centrifuged at 8000× *g* for 5 min, with the supernatant then collected for use. The samples were charged onto a Waters ACQUITY UPLC HSS T3 C18 column (Waters, USA) at 40 °C with 0.4 mL/min. We used the mobile phases of acidified water phase A (0.04% acetic acid) and acidified acetonitrile phase B (0.04% acetic acid). The analysis of the effluent was carried out using an ESI-triple quadrupole-linear ion trap, and the linear ion trap and triple quadrupole scans were acquired from the AB Sciex QTRAP6500 System.

The targeted metabolomic analyses were performed using previously published standard operating procedures [24]. The concentration and peak area of the standards were utilized to create a standard curve in which the sample concentrations were calculated. The steroid hormones were quantitated by comparison with the peaks areas of the external standards [25], then MultiQuant software version 2.1 (AB SCIEX, Framingham, MA, USA) was used for chromatogram review and peak area integration. For screening of the differential metabolites, we used the T-test as a univariate analysis. Those with a *T*-test *p*-value < 0.05 were regarded as differential steroid hormones.

### 2.6. Culture and Treatment of phGCs and phTCs

Granulosa cells from prehierarchical follicles (phGCs, from SYFs) were harvested following the previously described methods [26,27]. The phGCs were cultured at 37 °C, 5% CO_2_ with Medium 199 (Gibco, Grand Island, NY, USA), 5% fetal bovine serum (FBS, Gibco, USA), and 1% penicillin/streptomycin (Gibco, USA). Theca cells from pre-hierarchical follicles (phTCs, from SYFs) were collected according to previous reports [26,28,29]. The phTCs were cultured with DMEM/F12 (Gibco, USA) and supplemented with 10% FBS and 1% penicillin/streptomycin. A trypan blue stain was used to determine the cell viability, and then an automated cell counter (Countstar IC1000, Shanghai, China) was used to test the cell viability. After cell adhesion, the phGCs and phTCs were cultured with 1% DMSO (0 nM) or two concentrations of calcitriol (Med Chem Express, Monmouth, NJ, USA) (10 or 100 nM).

### 2.7. Cell Proliferation, Apoptosis, and Cell Cycle

The cell proliferation was assessed by using Cell Counting Kit8 (CCK-8, GLPBIO, Montclair, CA, USA) as per the manufacturer’s instructions. The cells were seeded in 100 μL of fresh medium at a density of 5000 cells/well in 96-well plates treated with 1% DMSO (0 nM) or two concentrations of VitD_3_ (10, or 100 nM). At the culture time of 12, 24, 36, and 48 h, each well was added to 10 μL of CCK8 reagent and incubated in a humidified atmosphere of 5% CO_2_ at 37 ℃ for 2 h. Absorption at 450 nm was measured using an Enspire Microplate Reader (PerkinElmer, Singapore).

The cell apoptosis was analyzed with the AnnexinV-FITC/PI apoptosis detection kit (KeyGen BioTech, Nanjing, China), following the manufacturer’s instructions. Cells were seeded at 50,000 cells/well in 6-well plates, and after 24 h of VitD_3_ treatment (0, 10, or 100 nM), the harvested cells were fixed with 500 μL binding buffer. Then, cells were treated with 5 μL annexin V-FITC and 5 μL propidium iodide in darkness for 15 min and analyzed for apoptosis using flow cytometry (Beckman Coulter FC500, Brea, CA, USA).

The cell cycle was detected by flow cytometry using the Cell Cycle Detection Kit (KeyGen BioTech, Nanjing, China). After various concentrations of VitD_3_ (0, 10, and 100 nM) were applied for 24 h, the cells were harvested, centrifuged, and re-suspended in PBS. The harvested cells were then stained with propidium iodide (PI) solution (50 µg/mL PI and 100 µg/mL RNase A with PBS) and cell cycle assayed by flow cytometry (Beckman Coulter FC500). A minimum of 10,000 cells were analyzed for each sample. Cell apoptosis and the cell cycle were analyzed using Flowjo_10.

### 2.8. Immunohistochemistry and Immunofluorescence Detection of VDR

The immunohistochemistry was performed according to a previously described method [30,31]. VDR rabbit pAb (A2194; ABclonal, Wuhan, China) was used as a primary antibody, and HRP goat antirabbit IgG (ab150182; Abcam, Cambridge, UK) was used as a secondary antibody. As for the immunofluorescence, cells were seeded onto coverslips in 12-well plates and VitD_3_-treated for 24 h. Then, the cells were fixed with 4% paraformaldehyde at room temperature for 20 min and immersed 3 times in cold PBS. The cells were then incubated with blocking buffer and then incubated with VDR rabbit pAb (A2194; ABclonal) that was diluted 1:100 as the primary antibody at 4 °C overnight. HRP goat antirabbit IgG was used as a secondary antibody (ab150182; Abcam). The nuclei were stained with DAPI (Sigma, USA) for 5 min. The images were obtained using fluorescence microscopy (Olympus BX53, Tokyo, Japan), and the intensity of the VDR was calculated using Image J software version 153.

### 2.9. Statistical Analysis

Our data were expressed as means ± standard error (SEM). After the homogeneity test of variance (Levene’s test), with VitD_3_ treatment as the main effect, the results were analyzed by one-way analysis of variance (ANOVA) followed by post hoc multiple comparisons (Duncan test), and *p*-value < 0.05 was considered as significant among groups. SPSS19.0 software (SPSS Inc., Chicago, IL, USA) was used to perform statistical analyses, and GraphPad Prism 8.0 (GraphPad Software Inc., San Diego, CA, USA) was also used for drawing figures.

## 3. Results

### 3.1. Effects of VitD_3_ on Follicle Number and SYF Structure of Layers

**Figure 1:**
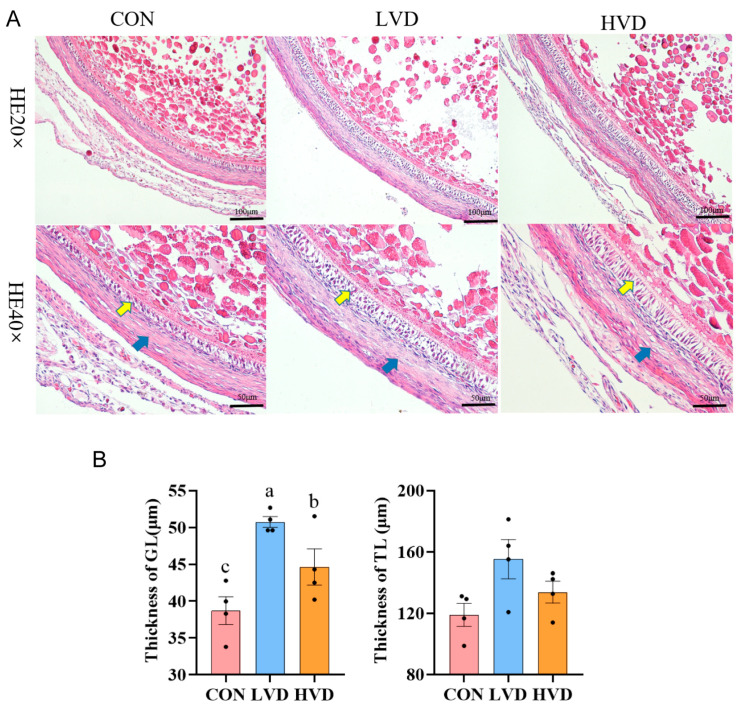
Effect of VitD_3_ on follicle development of young layers. (**A**) Follicular morphology in SYFs of VitD_3_-treated young layers. H&E staining was used to evaluate the morphology of SYFs. Yellow arrows indicate granulosa layers (GL), and blue arrows indicate theca layers (TLs). Scale bars: 100 (20×) and 50 μm (40×). (**B**) Summary of GL and TL thickness in SYFs. Values were the mean ± SEM (*n* = 4); bars with no common lowercase letters (a–c) differ significantly (*p* < 0.05). CON: with calcitriol 0 μg/kg BW; LVD: with calcitriol 10 μg/kg BW; HVD: with calcitriol 100 μg/kg BW; VitD_3_: 1α,25-dihydroxyvitamin D_3_.

The effects of VitD_3_ on the development of ovarian follicles in young layers, including preovulatory follicles (POFs), LYFs, and SYFs, are presented in Table 1. Although there were no differences observed in the number of POFs among treatments, low-dosage VitD_3_ (LVD) had a higher total number of LYFs, and high-dosage VitD_3_ (HVD) had a higher total number of SYFs compared to the control (CON) (*p* < 0.05). HE staining of the SYFs showed that the granulosa layers (GL) of LVD and HVD were significantly thicker than the control (CON) (*p* < 0.05), with the highest GL thickness in LVD, and a non-significant difference was found in the thickness of the theca layers (TLs). This demonstrated that the follicles exhibited an integral structure with clear boundaries between GL and TL and closely arranged GCs, and the TL structure of HVD became sparse (Figure 1A,B). These results hinted that a low dose of VitD_3_ might promote GC proliferation and increase the number of SYFs, both of which could influence follicle selection dynamics in laying hens.

### 3.2. VitD_3_ Promotes Expression of Genes Related to Steroidogenesis in SYFs

**Figure 2:**
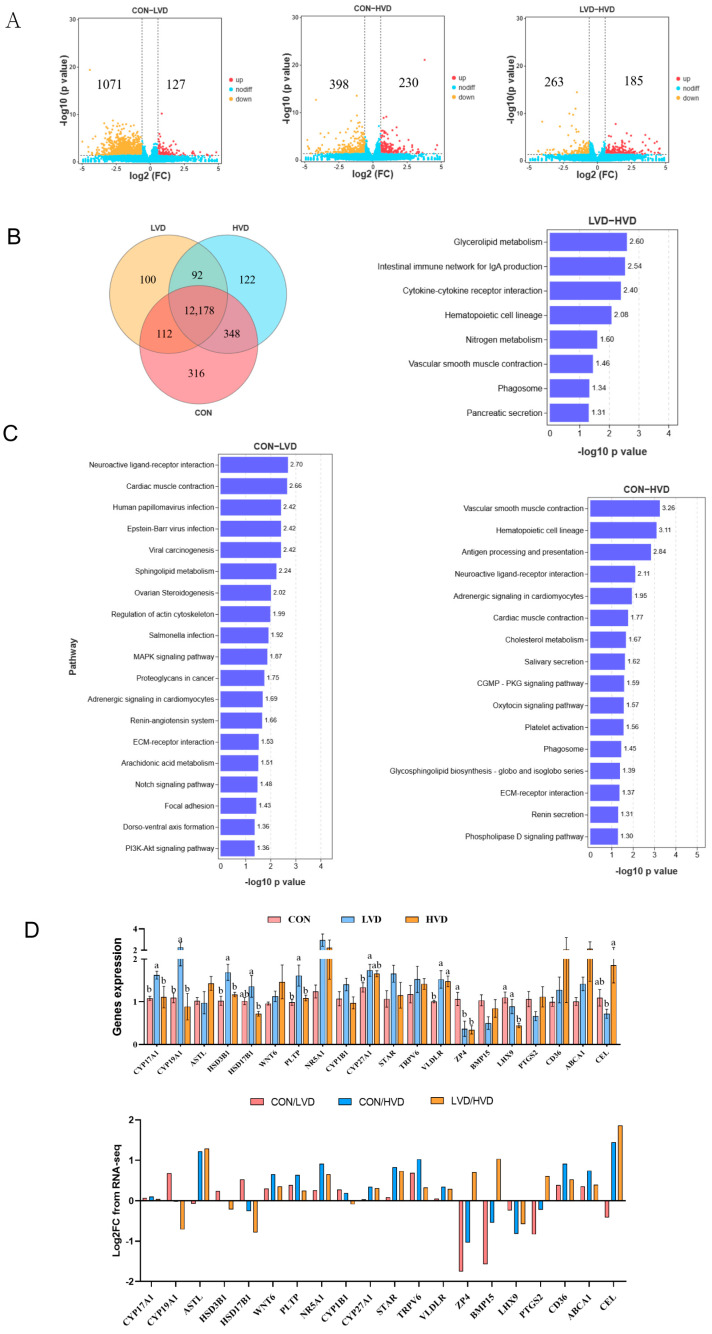
Small yellow follicle (SYF) transcriptome analyses reveal mRNA expression changes. (**A**) Volcano plot of differentially expressed genes (DEGs) between VitD_3_-treated and control in SYFs. Red spots and yellow spots represent significantly upregulated and downregulated genes, respectively (*p*-value < 0.05 and fold change > 1.5). Blue spots: no difference in gene expression. (**B**) Venn diagram of DEGs among groups. (**C**) Kyoto Encyclopedia of Genes and Genomes (KEGG) analysis of DEGs between CON and LVD, CON and HVD, and LVD and HVD. The significantly enriched KEGG pathways of the three groups are shown; *p*-value < 0.05 was used as the threshold of significant enrichment for KEGG. (**D**) Relative mRNA expression and RNA-seq data of the 20 selected DEGs. The mRNA expression values shown are means ± SEM (*n* = 5); bars with no common lowercase letters (a, b) differ significantly (*p* < 0.05). CON: with calcitriol 0 μg/kg BW; LVD: with calcitriol 10 μg/kg BW; HVD: with calcitriol 100 μg/kg BW; VitD_3_: 1α,25-dihydroxyvitamin D_3_.

To screen the differentially expressed genes (DEGs) due to VitD_3_ treatment, RNA-seq was utilized for the SYFs among the CON, LVD, and HVD. We visualized significant changes in the mRNA expression of the VitD_3_-treated groups using a volcano plot and Venn diagram (Figure 2A,B). In the CON vs. LVD sequencing, 1198 DEGs were detected, including 127 upregulated genes and 1071 downregulated genes. In the CON vs. HVD sequencing, 628 DEGs were detected, including 230 upregulated genes and 398 downregulated genes. In the LVD vs. HVD sequencing, 448 DEGs were detected, including 185 upregulated genes and 263 downregulated genes. The KEGG pathway enrichment analysis of the DEGs revealed that they were notably enriched in functional pathways that are critical in follicle development, such as ovarian steroidogenesis, cholesterol metabolism, glycerolipid metabolism, ECM–receptor interaction, and the MAPK pathway and PI3K–AKT–signaling pathways (Figure 2C). In addition, compared with the CON, *CYP19A1* was significantly upregulated, and *BMP15*, *ZP4*, and *PTGS2* were significantly downregulated in LVD; *CYP27A1*, *CD36*, and *VLDLR* were significantly upregulated, and *ZP4* and *LHX9* were significantly downregulated in HVD (Supplementary Table S2). To verify the transcriptomic data, 20 genes in ovarian steroidogenesis and cholesterol metabolism were selected for RT-qPCR, which showed that the regulation trend of the relative mRNA expression was consistent with the results obtained from the transcriptomic analysis (Figure 2D).

### 3.3. Steroid Hormone Content in SYF Fluid and Serum Changed after VitD_3_ Treatment

**Figure 3:**
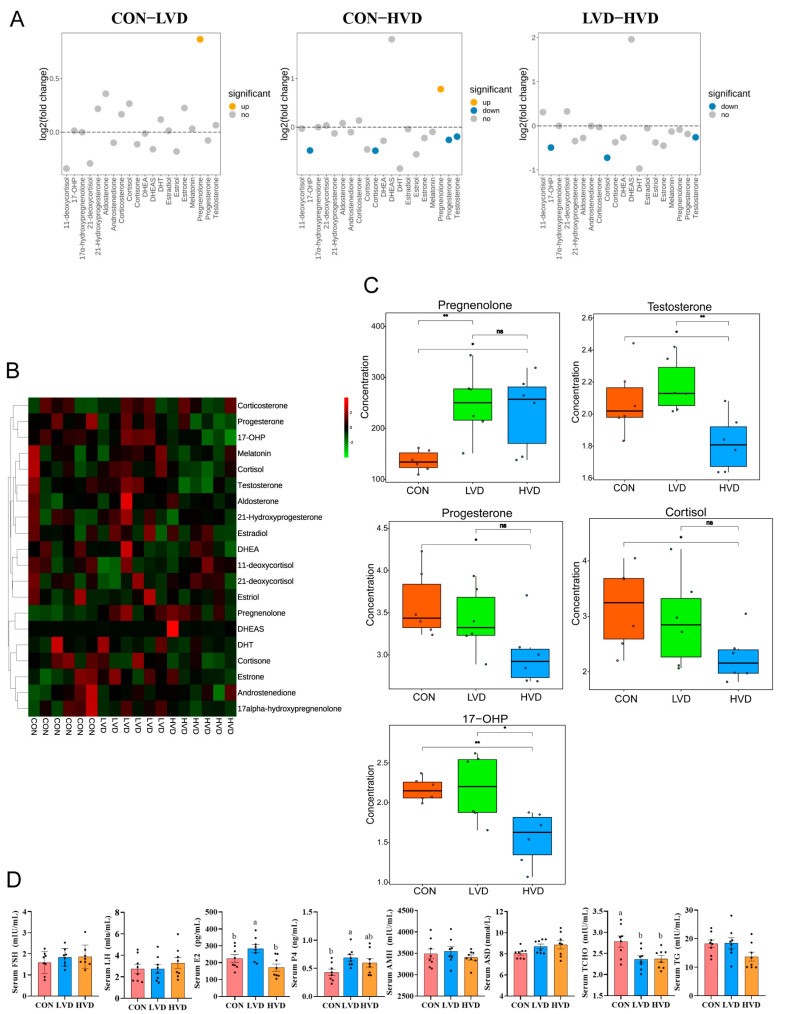
VitD_3_ altered steroid hormone contents in young layers’ SYF fluid and serum. (**A**) The fold change distribution of 20 steroid hormones among the three groups. (fold change distribution of 20 steroid hormones in CON vs. LVD; fold change distribution of 20 steroid hormones in LVD vs. HVD; fold change distribution of 20 steroid hormones in CON vs. HVD; *n* = 6). (**B**) Heat map of 20 differential steroid hormones quantified in SYFs fluid across the three groups. The *X*-axis represents different groups, and the *Y*-axis represents the steroid hormones. (**C**) VitD_3_ significantly alters five steroid hormones in chicken SYF fluid. Boxplot of five differential steroid hormones including testosterone, pregnenolone, 17-hydroxyprogesterone (17-OHP), cortisol, and progesterone in chicken SYF fluid. Steroid-hormone-targeted metabolomics profiling examined in chicken SYFs fluid (*n* = 6, * *p* < 0.05, ** *p* < 0.01, ns not significant). (**D**) Effect of VitD_3_ treatment on steroid hormone content and cholesterol metabolism index in young layers’ serum. ELISA assay of serum steroid hormones follicle-stimulating hormone (FSH), luteinizing hormone (LH), estradiol (E2), progesterone (P4), androstenedione (ASD), antimullerian hormone (AMH), and cholesterol metabolism index triglycerides (TGs), and total cholesterol (TCHO). Values are expressed as means ± SEM (*n* = 8), and bars with no common lowercase letters (a, b) differed significantly (*p* < 0.05). CON: with calcitriol 0 μg/kg BW; LVD: with calcitriol 10 μg/kg BW; HVD: with calcitriol 100 μg/kg BW; VitD_3_: 1α,25-dihydroxyvitamin D_3_.

To further verify the effects of VitD_3_ on steroid hormones in SYF, steroid-hormone-targeted metabolomics was performed and identified 20 steroid hormones (Supplementary Figure S1). Among these, 11 steroid hormones exhibited increased levels in LVD and decreased levels in HVD, 5 of which were significantly different in SYF fluid after VitD_3_ treatment (*p* < 0.05), namely testosterone, pregnenolone, 17-hydroxyprogesterone (17-OHP), cortisol, and progesterone (Figure 3A–C). Pregnenolone was significantly upregulated in both LVD and HVD, whereas four steroid hormones were significantly decreased in HVD.

In serum, there was also a significant difference in estradiol (E2), progesterone (P4), and total cholesterol (TCHO) among the different treatments (Figure 3D). Compared with the CON, the E2 and P4 concentration was significantly increased in LVD (*p* < 0.05), and the HVD did not show a difference. In addition, the serum TCHO content from LVD and HVD was lower than the CON (*p* < 0.05). These results showed that, in serum, VitD_3_ treatment significantly increased E2 and P4, whereas it decreased the TCHO content (*p* < 0.05).

### 3.4. VitD_3_ Promoted the Proliferation and Cell Cycle of Granulosa Cells from Pre-Hierarchical Follicles (phGCs) and Theca Cells from Pre-Hierarchical Follicles (phTCs) and Inhibited Their Apoptosis

**Figure 4:**
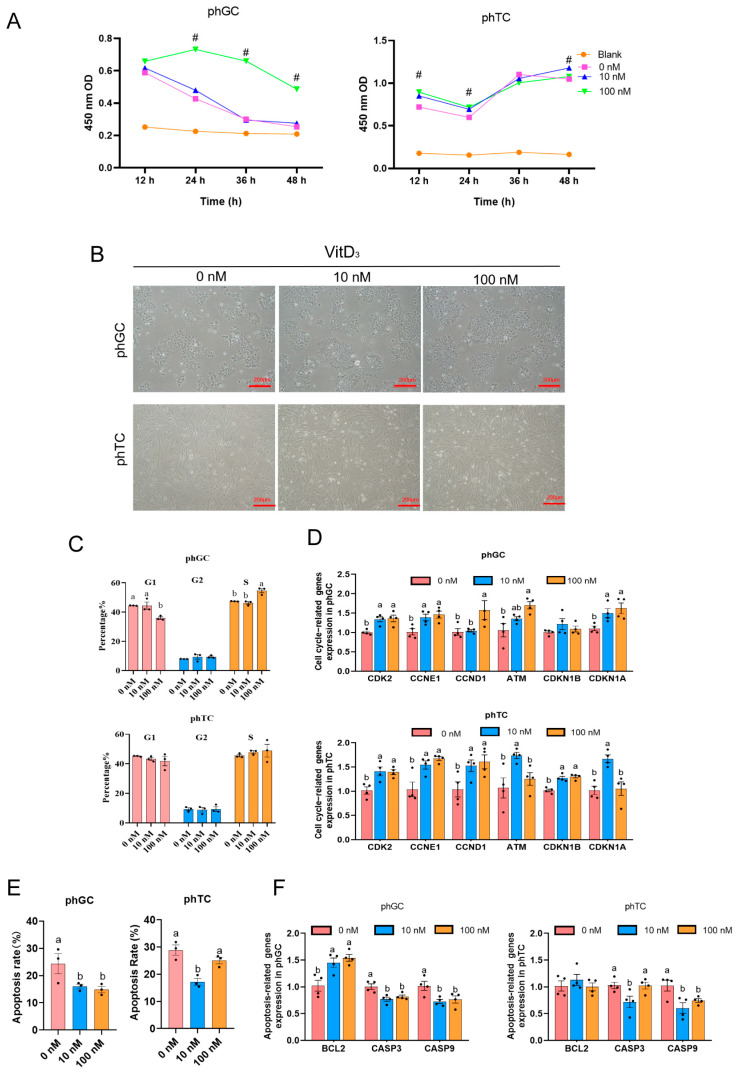
Effect of VitD_3_ on phGCs, phTCs proliferation, cell-cycle progression, and apoptosis. (**A**) Effect of VitD_3_ on phGC and phTC proliferation (*n* = 6, # *p* < 0.05). Cell proliferation was detected by an CCK8 assay cultured at 12, 24, 36, and 48 h. (**B**) Pictures of cultured phGCs and phTCs. Scale bars: 200 μm. (**C**) Flow cytometric analysis of cell cycle in phGCs and phTCs after VitD_3_ treatment. Percentage of phGCs and phTCs in different phases of the cell cycle (G1, G2, S). Quantization of phGCs and phTCs at different stages (*n* = 3). Cultured phGCs and phTCs were treated with VitD_3_ at 0, 10, and 100 nM for 24 h. (**D**) VitD_3_ altered the mRNA expression of cell-cycle-related genes. RT-qPCR was performed to examine changes in mRNA levels of genes involved in cell cycle (*CDK2*, *CCNE1*, *CCND1*, *ATM*, *CDKN1A*, and *CDKN1B*) in phGCs and phTCs (*n* = 4). (**E**) Apoptosis was assessed in phGCs and phTCs by flow cytometry after treatment with VitD_3_ and the quantification of apoptotic cells (*n* = 3). (**F**) mRNA levels of genes related to apoptosis (*BCL2*, *CASP3*, and *CASP9*), obtained by RT-qPCR of exposure to VitD_3_ (*n* = 4). Values are the mean ± SEM; bars with no common lowercase letters (a, b) differ significantly (*p* < 0.05). phGCs: granulosa cells from prehierarchical follicles; phTCs: theca cells from prehierarchical follicles; VitD_3_: 1α,25-dihydroxyvitamin D_3_.

To further verify the function of VitD_3_ in follicular development, phGCs and phTCs were isolated to investigate the effect of VitD_3_ on cell proliferation, cell-cycle progression, and apoptosis, and the cultured phGCs and phTCs are presented in Figure 4B. VitD_3_ increased phGC cell proliferation (*p* < 0.05; Figure 4A). To further confirm the effect of VitD_3_ on cell proliferation, we examined the cell cycle using flow cytometric analysis and the mRNA expression of cell-cycle-related genes after VitD_3_ treatment. It was shown that for phGCs, VitD_3_ (100 nM) treatment significantly increased cells in the S-phase, accompanied by a reduction in the G1-phase compared with 0 nM. Consistently, the mRNA expression levels of the cell-cycle-related genes *CDK2*, *CCNE1*, *CCND1*, *ATM*, and *CDKN1A* in phGCs were elevated by VitD_3_ (100 nM) (*p* < 0.05; Supplementary Figure S2A and Figure 4C,D). As shown in Figure S2B and Figure 4E,F, VitD_3_ (10–100 nM) treatment could significantly inhibit apoptosis rates in phGCs. RT-qPCR showed that the mRNA levels of *CASP3* and *CASP9* in the phGCs was significantly decreased by VitD_3_ (10–100 nM) treatment, and the *BCL2* was significantly increased (*p* < 0.05).

Furthermore, the proliferation of phTCs increased in response to VitD_3_ at 100 nM within 24 h, and the VitD_3_ at 10 nM at 48 h showed a higher absorbance (*p* < 0.05; Figure 4A). However, our results showed that VitD_3_ had no significant effect on the cell-cycle progression of phTCs, but it increased the mRNA expression of all six cell-cycle-related genes at VitD_3_ (10 nM) (*p* < 0.05; Supplementary Figure S2A and Figure 4C,D). Similarly, 10 nM VitD_3_ treatment was able to significantly inhibit the apoptosis rates in phTCs, and the mRNA levels of *CASP3* and *CASP9* significantly decreased (*p* < 0.05; Supplementary Figure S2B and Figure 4E,F). Generally, our results showed that 10–100 nM VitD_3_ had a clear effect on promoting the cell proliferation and positively promoted the synthesis of DNA, inhibiting apoptosis, especially in phGCs.

### 3.5. VitD_3_ Enhances Steroid Hormone Biosynthesis in phGCs and phTCs

**Figure 5:**
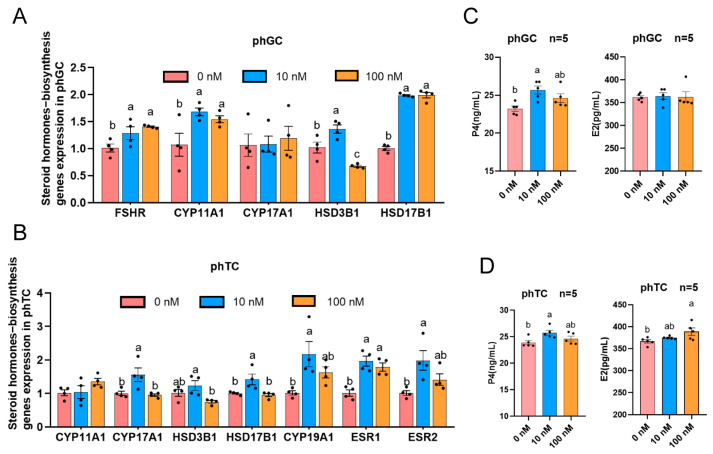
VitD_3_ alters steroid hormone biosynthesis in granulosa cells from prehierarchical follicles (phGCs) and theca cells from prehierarchical follicles (phTCs). (**A**) mRNA expression of steroid hormone-biosynthesis genes in phGCs (*FSHR*, *CYP11A1*, *CYP17A1*, *HSD3B1*, *HSD17B1*; *n* = 4). (**B**) mRNA expression of steroid hormone-biosynthesis genes in phTCs (*CYP11A1*, *CYP17A1*, *HSD3B1*, *CYP19A1*, *HSD17B1*, *ESR1*, *ESR2*; *n* = 4). mRNA expression levels of these genes were analyzed by real-time PCR. (**C**) Effect of VitD_3_ on progesterone (P4), and estradiol (E2) production in phGCs (*n* = 5). (**D**) Effect of VitD_3_ on P4 and E2 production in phTCs (*n* = 5). P4 and E2 concentrations in cell medium were analyzed by ELISA assay. Values are the mean ± SEM. Bars with no common lowercase letters (a, b) differ significantly (*p* < 0.05). phGCs: granulosa cells from prehierarchical follicles; phTCs: theca cells from prehierarchical follicles; VitD_3_: 1α,25-dihydroxyvitamin D_3._

To examine the direct actions of VitD_3_ in regulating steroid hormone biosynthesis, the mRNA levels of the genes related to steroid hormone biosynthesis were tested. The levels of mRNA for *FSHR*, *CYP11A1*, and *HSD17B1* were upregulated in 10 nM VitD_3_ when compared to the control, while 100 nM VitD_3_ downregulated the *HSD3B1* in phGCs (*p* < 0.05; Figure 5A). Moreover, the treatment of phTCs with 10 nM VitD_3_ upregulated the expression of most of the genes tested compared with the control (Figure 5B). To further confirm these results, the contents of P4 and E2 in the culture media were tested. After exposure for 24 h, the P4 levels in phGCs and phTCs treated with 10 nM VitD_3_ were significantly higher than those in the control, and 100 nM VitD_3_ significantly increased E2 in phTC medium in a dose-dependent manner (*p* < 0.05), with no significant effect on E2 content in phGC (Figure 5C,D). Consistent with the results of the steroid hormone-biosynthesis enzyme mRNA expression, VitD_3_ increased the production of steroid hormones biosynthesis in cell-cultured medium compared with the control.

### 3.6. VitD_3_ Promotes VDR Expression in phGCs and phTCs

**Figure 6:**
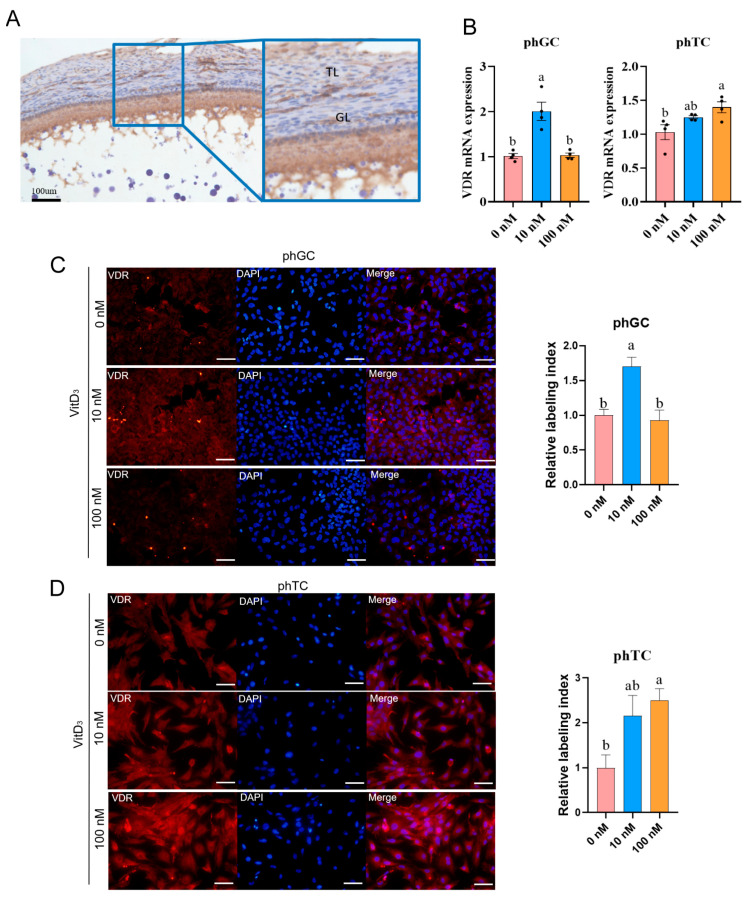
VitD_3_ stimulated chicken phGCs and phTCs VDR expression. (**A**) Immunohistochemical staining of VDR in the chicken small yellow follicle (SYF). Scale bar: 100 μm (**B**) *VDR* mRNA expression after treatment with VitD_3_ in phGCs and phTCs (*n* = 4). (**C**) Immunofluorescence assay for VDR in phGCs after 48 h culture and the relative VDR index in different treatment (*n* = 3). (**D**) Immunofluorescence assay for VDR in phTC and the relative VDR index in different treatment (*n* = 3). Values are the mean ± SEM; bars with no common lowercase letters (a, b) differ significantly (*p* < 0.05). Granulosa layers (GL), theca layers (TL); phGCs: granulosa cells from prehierarchical follicles; phTCs: theca cells from prehierarchical follicles; VitD_3_: 1α,25-dihydroxyvitamin D_3_; VDR: vitamin D receptor.

The localization of VDR is shown by immunohistochemistry in SYFs, and VDR was present in GL and TL (Figure 6A). When phGCs were treated with 10 nM VitD_3_, the mRNA expression of *VDR* was significantly higher than for 0 nM. In addition, 100 nM VitD_3_ significantly upregulated phTCs’ *VDR* mRNA expression and exhibited dose dependence (*p* < 0.05; Figure 6B). A similar trend was observed in the immunofluorescence assay, where VitD_3_ significantly activated the relative labeling index of VDR in both phGCs and phTCs (Figure 6C,D).

## 4. Discussion

### 4.1. VitD_3_ Regulates the Development of PHFs in Young Layers

VitD_3_ participates in many biological processes, including cell growth and differentiation, immune function, regulation of cardiovascular function, and hormone metabolism and has pivotal roles in maintaining calcium and phosphorus homeostasis [32]. The functions of VitD_3_ are not confined to the maintenance of bone health and mineral metabolism; it also plays an important role in follicle development in mammals [6,7,33,34]. Nevertheless, the mechanism of VitD_3_ in regulating follicle development remains unknown. Thus, these in vitro studies were carried out in the absence of direct evidence for the roles played by VitD_3′_. Our study revealed the effect of VitD_3_ on the follicle development of young layers in vivo and in vitro; we found that the number of PHFs in VitD_3_-treated hens was significantly higher than those in the control, and treatment with VitD_3_ promoted steroid biosynthesis and cell proliferation.

It is widely known that regular granulosa cell proliferation and apoptosis support the development of follicles in mammals [35,36]. Previous studies showed that VitD_3_ can indirectly affect follicle development in goat luteinized granulosa cells and chicken follicle GCs in vitro [34,37]. Research in macaques found that VitD_3_ is associated with higher pregnancy rates by significantly enhancing the cell proliferation and differentiation of GCs [7]. Various studies also indicated an alteration in cell apoptosis in mammals. In our study, VitD_3_ supplementation significantly increased the number of PHFs and GL thickness in hens, promoted cell proliferation, and inhibited apoptosis in in vitro cultured follicle cells. Therefore, these results provide a possible mechanism for the follicle selection that VitD_3_ supplementation contributes to PHF development by promoting cell proliferation and inhibiting apoptosis. However, studies also showed that VitD_3_ supplementation had an inhibitory effect on cancer cell proliferation and a likely benefit in colon and breast cancer [38,39]. Consequently, the effect of VitD_3_ on cellular responses might depend on the different cell types.

### 4.2. VitD_3_ Promotes Steroid Hormone Synthesis in the Follicles of Layers

Follicular development is a complex process regulated by multiple genes and steroid hormones. Steroid syntheses play an essential role in follicular development, and these concentrations and activities in the animal body are tightly regulated by the steroid biosynthesis pathway [40]. In addition, *CYP11A1*, *CYP17A1*, *CYP19A1*, *HSD3B1*, and *HSD17B1* are key members involved in steroid biosynthetic processes. *CYP11A1* is the first step of steroidogenesis and catalyzes the conversion of cholesterol to pregnenolone, and the gene controls the rate of synthesis of all steroid hormones. *CYP17A1*, a steroidogenic enzyme, acts as the enzymatic gateway to glucocorticoid and androgen synthesis [41]. Aromatase is encoded by *CYP19A1*, a critical enzyme catalyzing estrogen biosynthesis [42]. In this study, the KEGG results showed that ovarian steroidogenesis, cholesterol metabolism, glycerolipid metabolism, ECM–receptor interaction, the MAPK pathway, and PI3K-AKT-signaling pathways, which are involved in follicle development and oocyte maturation [14,43], were significantly enriched after VitD_3_ treatment. Several important pathways were enriched in the low-dose VitD_3_ compared with CON, including ovarian steroidogenesis, ECM–receptor interaction, the MAPK pathway, and PI3K–AKT signaling, and the high-dose VitD_3_ enriched the cholesterol metabolism, ECM–receptor interaction, and vascular smooth muscle contraction. This is particularly interesting in light of the fact that the different doses of VitD_3_ affected the pathways in different ways; the LVD vs. HVD enriched the glycerolipid metabolism, cytokine–cytokine receptor, nitrogen metabolism, and vascular smooth muscle contraction, which may be related to the negative feedback regulation of hormones or different doses of VitD_3_ mediating cell functions through genomic and non-genomic mechanisms [44,45]. Meanwhile, the significantly upregulated expression of steroid hormone biosynthesis-related genes (*CYP19A1*, *HSD3B1*, and *HSD17B1*) was found both in vivo and in vitro after VitD_3_ treatment. Collectively, these findings indicated that VitD_3_ stimulates steroid hormone synthesis at the transcriptional level.

Hormones synthesized from steroidogenesis include testosterone, progesterone, and estrogen [46]. Testosterone, progesterone, and estrogen play critical functions in follicle development, and progesterone is used to synthesize testosterone and estrogen. Through targeted metabolomics profiling, our research found that five hormones, testosterone, pregnenolone, 17–OHP, cortisol, and progesterone, significantly differed in layers’ follicle fluid, and pregnenolone was significantly upregulated in both VitD_3_ doses. The other four hormones were significantly lower at higher doses. Pregnenolone is a steroid hormone involved in the steroidogenesis of progesterone, androgens, and estrogens and is synthesized from cholesterol in the mitochondrion [47]. In previous studies, testosterone has been proved to stimulate follicle aggregation, growth, and development and may enhance oxygen supply to GCs and energy metabolism of ovarian follicles [48,49]. The enzyme *HSD17B1* can further convert progesterone to testosterone [50]. In our study, VitD_3_ was shown to fuel testosterone production at low doses and inhibit it at high doses in follicle fluid, and the expressional tendency of *HSD17B1* mRNA demonstrated a similar trend in SYFs. We conclude that VitD_3_ may directly modulate pregnenolone and testosterone metabolism.

There is increasing evidence of the benefits of VitD_3_ in mammal steroid hormone biosynthesis in vitro cultured cells. Studies on human skin fibroblasts showed that incubation with VitD_3_ increased the expression of *CYP19A1* and promoted the synthesis of E2 [51,52]. VitD_3_ treatment led to an increase in E2 production in in vitro cultured human ovarian GCs and in porcine ovarian GCs [33]. *CYP19A1* was expressed in the GL and TL of the mammal follicles, whereas it was only expressed in the chicken TL. The estrogen in chicken follicles is mainly derived from TCs, while GCs are the source of P4 [53]. Therefore, the regulation of VitD_3_ on GCs and TCs was further examined in our in vitro study, finding that the mRNA expression of steroid hormone biosynthesis-related genes was upregulated in the VitD_3_–treated samples. Furthermore, the levels of E2 and P4 in serum and in cell-cultured medium changed with VitD_3_ treatment, indicating that VitD_3_ regulates the synthesis of E2 and P4, as in mammals. Cholesterol is an essential precursor of steroidogenesis; the steroidogenic cells derive cholesterol through de novo synthesis and through the uptake of plasma-derived cholesterol [54]. In this study, a significant decrease in serum TCHO occurred after VitD_3_ intervention, suggesting that the consumption of TCHO for hormone synthesis might be accelerated. These results supported our hypothesis that VitD_3_ regulates steroid hormone biosynthesis by directly enhancing cholesterol transport converted into pregnenolone and indirectly increasing estrogen synthesis in the chicken follicles.

### 4.3. VitD_3_ Triggers VDR Expression In Vitro Cultured Follicle Cells

VitD_3_ exerts its functions through VDR, which is present in various animal tissues [55]. Other studies demonstrated the presence of VDR in the placenta, uterus, ovaries, and granulosa cells of humans and rodents and in the male testes, epididymis, and sperm cells, and it is also expressed in chicken GCs [37,56,57,58,59,60]. In this research, we found, for the first time, that VDR was present both in the TL and GL in SYFs, and the VDR present in TL has not been reported in hens. Furthermore, the treatment of low-dose VitD_3_ markedly increased the thickness of the GL, and the expression of steroid hormone-biosynthesis related genes was significantly increased in SYFs. These data indicate that VitD_3_ stimulates the proliferation of phGCs and phTCs in SYFs to increase the thickness of GL and fails to increase the thickness of TL, which may be related to the location and number of VDR. The VDR expression in follicles suggests its importance in follicle development [61] and is essential for the growth of GCs and TCs of PHFs in hens.

In the invariant natural killer T (INKT) cells, it was found that when VDR was knocked out, the cells could not proliferate and mature normally [62]. When the VDR was knocked down, a significant inhibiting effect on tube formation was observed [63]. Additionally, VDR knockout mice were infertile and had decreased folliculogenesis and *CYP19A1* expression, whereas after supplementing with E2, the uterine weight increased [64,65]. Therefore, VDR might be involved in follicle development and may play a role in cell proliferation. Our results showed that VitD_3_ could promote phGC and phTC proliferation by increasing cell cycling and suppressing cell apoptosis by increasing *BCL2* expression and decreasing *CASP3* and *CASP9* expression. Two types of follicle cell showed differences in the sensitivity to VitD_3_, leading to diverse cellular responses, including cell proliferation, cell cycling, and apoptosis at 10–100 nM VitD_3_ levels. Taken together, these results suggest that VitD_3_ increased cell proliferation, inhibited cell apoptosis, and induced the expression of VDR in SYFs, which may have similar functions in mammals. Thus, further studies are warranted to investigate the mechanism by which VitD_3_ mediates cellular effects and to delve more deeply into its autocrine function using in vitro follicle culture.

## 5. Conclusions

**Figure 7:**
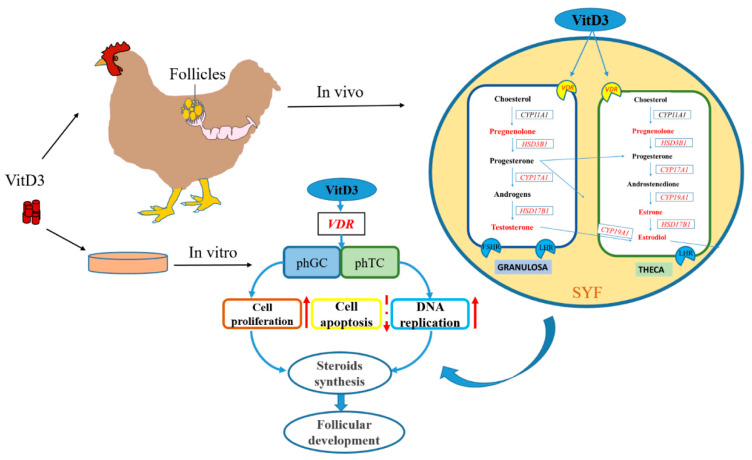
The regulatory pattern of VitD_3_ on follicular development in young layers’ PHFs. The solid red arrows represent promotion, whereas the dashed red arrows represent inhibition. The red genes and steroid hormones represent significant regulation by VitD_3_.

In conclusion, our results indicated that VDR is expressed in young layers’ GCs and TCs, and VitD_3_ regulated steroidogenesis (*CYP19A1*, *CYP17A1* and *HSD3B1*) and the production of testosterone, estradiol, and progesterone in chicken PHFs. Furthermore, VitD_3_ increased the GL thickness and participated in follicle cell function by upregulating proliferation, enhancing cell-cycle progression, and inhibiting apoptosis. These results suggest that VitD_3_ might affect follicle development by promoting follicle cell function and steroid hormone biosynthesis. Additionally, VitD_3_ treatment showed a direct action on the PHFs and is likely to participate in the follicle selection process, which may provide a better environment for follicular development in young layers (Figure 7). This research is of benefit as it helps to clarify the mechanism of VitD_3_ on PHF development and steroid hormones biosynthesis in the young layer model.

## Figures and Tables

**Table 1:** Effect of VitD_3_ treatment on follicle number of POFs, LYFs, and SYFs in young layers.

Items	Groups			SEM	*p*-Value
	CON	LVD	HVD		
Number of POFs	5.15	5.20	5.35	0.09	0.637
Number of LYFs	4.95 ^b^	6.45 ^a^	4.00 ^b^	0.32	0.006
Number of SYFs	5.15 ^b^	5.90 ^ab^	6.65 ^a^	0.25	0.046

Preovulatory follicles (POFs, F1–F6), large yellow follicles (LYFs, 9–10 mm), small yellow follicles (SYFs, 6–8 mm). CON: with calcitriol l 0 μg/kg BW, LVD: with calcitriol 10 μg/kg BW, HVD: with calcitriol 100 μg/kg BW. VitD_3_: 1α,25-dihydroxyvitamin D_3_. ^a,b^ Values with no common lowercase letters differ significantly (*p* < 0.05; *n* = 20).

## Data Availability

Datasets used in this study are available from the corresponding author on reasonable request. The sequencing datasets presented in this study are submitted in the Genome Sequence Archive in the BIG Data Center under the BioProject: PRJCA013159.

## Supplementary Materials

The following supporting information can be downloaded at: https://www.mdpi.com/article/10.3390/cimb45050256/s1.

## References

[1] Świątkiewicz S., Arczewska-Włosek A., Bederska-Lojewska D., Józefiak D. (2019). Efficacy of dietary vitamin D and its metabolites in poultry—Review and implications of the recent studies. World’s Poult. Sci. J..

[2] Whitehead C.C. (2004). Overview of bone biology in the egg-laying hen. Poult. Sci..

[3] Buell J.S., Dawson-Hughes B. (2008). Vitamin D and neurocognitive dysfunction: Preventing “D”ecline?. Mol. Asp. Med..

[4] Bar A., Vax E., Striem S. (1999). Relationships among age, eggshell thickness and vitamin D metabolism and its expression in the laying hen. Comp. Biochem. Physiol. Part A Mol. Integr. Physiol..

[5] Frost T.J., Roland D.A., Untawale G.G. (1990). Influence of vitamin D3, 1 alpha-hydroxyvitamin D3, and 1,25-dihydroxyvitamin D3 on eggshell quality, tibia strength, and various production parameters in commercial laying hens. Poult. Sci..

[6] Xu J., Lawson M.S., Xu F., Du Y., Tkachenko O.Y., Bishop C.V., Pejovic-Nezhat L., Seifer D.B., Hennebold J.D. (2018). Vitamin D3 Regulates Follicular Development and Intrafollicular Vitamin D Biosynthesis and Signaling in the Primate Ovary. Front. Physiol..

[7] Xu J., Hennebold J.D., Seifer D.B. (2016). Direct vitamin D3 actions on rhesus macaque follicles in three-dimensional culture: Assessment of follicle survival, growth, steroid, and antimullerian hormone production. Fertil. Steril..

[8] Parikh G., Varadinova M., Suwandhi P., Araki T., Rosenwaks Z., Poretsky L., Seto-Young D. (2010). Vitamin D regulates steroidogenesis and insulin-like growth factor binding protein-1 (IGFBP-1) production in human ovarian cells. Horm. Metab. Res..

[9] Lorenzen M., Boisen I.M., Mortensen L.J., Lanske B., Juul A., Jensen M.B. (2017). Reproductive endocrinology of vitamin D. Mol. Cell. Endocrinol..

[10] Fang F., Ni K., Cai Y., Shang J., Zhang X., Xiong C. (2017). Effect of vitamin D supplementation on polycystic ovary syndrome: A systematic review and meta-analysis of randomized controlled trials. Complement. Ther. Clin. Pract..

[11] Tosca L., Crochet S., Ferre P., Foufelle F., Tesseraud S., Dupont J. (2006). AMP-activated protein kinase activation modulates progesterone secretion in granulosa cells from hen preovulatory follicles. J. Endocrinol..

[12] Tilly J.L., Kowalski K.I., Johnson A.L. (1991). Stage of ovarian follicular development associated with the initiation of steroidogenic competence in avian granulosa cells. Biol. Reprod..

[13] Johnson A.L., Woods D.C. (2009). Dynamics of avian ovarian follicle development: Cellular mechanisms of granulosa cell differentiation. Gen. Comp. Endocrinol..

[14] Johnson A.L. (2015). Ovarian follicle selection and granulosa cell differentiation. Poult. Sci..

[15] Lovell T.M., Gladwell R.T., Groome N.P., Knight P.G. (2003). Ovarian follicle development in the laying hen is accompanied by divergent changes in inhibin A, inhibin B, activin A and follistatin production in granulosa and theca layers. J. Endocrinol..

[16] Schneider W.J. (2009). Receptor-mediated mechanisms in ovarian follicle and oocyte development. Gen. Comp. Endocrinol..

[17] Pertea M., Kim D., Pertea G.M., Leek J.T., Salzberg S.L. (2016). Transcript-level expression analysis of RNA-seq experiments with HISAT, StringTie and Ballgown. Nat. Protoc..

[18] Wang J., Zhao C., Li J., Feng Y., Gong Y. (2017). Transcriptome analysis of the potential roles of FOXL2 in chicken pre-hierarchical and pre-ovulatory granulosa cells. Comp. Biochem. Physiol. Part D Genom. Proteom..

[19] Chen S., Zhou Y., Chen Y., Gu J. (2018). fastp: An ultra-fast all-in-one FASTQ preprocessor. Bioinformatics.

[20] Kim D., Langmead B., Salzberg S.L. (2015). HISAT: A fast spliced aligner with low memory requirements. Nat. Methods.

[21] Anders S., Pyl P.T., Huber W. (2015). HTSeq—A Python framework to work with high-throughput sequencing data. Bioinformatics.

[22] Love M.I., Huber W., Anders S. (2014). Moderated estimation of fold change and dispersion for RNA-seq data with DESeq2. Genome Biol..

[23] Kanehisa M., Goto S. (2000). KEGG: Kyoto encyclopedia of genes and genomes. Nucleic Acids Res..

[24] Schoeman J.C., Harms A.C., van Weeghel M., Berger R., Vreeken R.J., Hankemeier T. (2018). Development and application of a UHPLC-MS/MS metabolomics based comprehensive systemic and tissue-specific screening method for inflammatory, oxidative and nitrosative stress. Anal. Bioanal. Chem..

[25] Gika H.G., Wilson I.D., Theodoridis G.A. (2014). LC-MS-based holistic metabolic profiling. Problems, limitations, advantages, and future perspectives. J. Chromatogr. B Anal. Technol. Biomed. Life Sci..

[26] Gilbert A.B., Evans A.J., Perry M.M., Davidson M.H. (1977). A method for separating the granulosa cells, the basal lamina and the theca of the preovulatory ovarian follicle of the domestic fowl (*Gallus domesticus*). J. Reprod. Fertil..

[27] Li J., Luo W., Huang T., Gong Y. (2019). Growth differentiation factor 9 promotes follicle-stimulating hormone-induced progesterone production in chicken follicular granulosa cells. Gen. Comp. Endocrinol..

[28] Tilly J.L., Johnson A.L. (1989). Regulation of androstenedione production by adenosine 3′,5′-monophosphate and phorbol myristate acetate in ovarian thecal cells of the domestic hen. Endocrinology.

[29] Guo Y., Cheng L., Li X., Tang S., Zhang X., Gong Y. (2022). Transcriptional regulation of CYP19A1 expression in chickens: ESR1, ESR2 and NR5A2 form a functional network. Gen. Comp. Endocrinol..

[30] Heidari B., Rahmati-Ahmadabadi M., Akhondi M.M., Zarnani A.H., Jeddi-Tehrani M., Shirazi A., Naderi M.M., Behzadi B. (2012). Isolation, identification, and culture of goat spermatogonial stem cells using c-kit and PGP9.5 markers. J. Assist. Reprod. Genet..

[31] Qi Z., Li Y., Zhao B., Xu C., Liu Y., Li H., Zhang B., Wang X., Yang X., Xie W. (2017). BMP restricts stemness of intestinal Lgr5^+^ stem cells by directly suppressing their signature genes. Nat. Commun..

[32] Bikle D.D. (2012). Vitamin D and the skin: Physiology and pathophysiology. Rev. Endocr. Metab. Disord..

[33] Hong S.H., Lee J.E., An S.M., Shin Y.Y., Hwang D.Y., Yang S.Y., Cho S.K., An B.S. (2017). Effect of Vitamin D3 on Biosynthesis of Estrogen in Porcine Granulosa Cells via Modulation of Steroidogenic Enzymes. Toxicol. Res..

[34] Yao X., Zhang G., Guo Y., Ei-Samahy M., Wang S., Wan Y., Han L., Liu Z., Wang F., Zhang Y. (2017). Vitamin D receptor expression and potential role of vitamin D on cell proliferation and steroidogenesis in goat ovarian granulosa cells. Theriogenology.

[35] Asselin E., Xiao C.W., Wang Y.F., Tsang B.K. (2000). Mammalian follicular development and atresia: Role of apoptosis. Neurosignals.

[36] Matsuda F., Inoue N., Manabe N., Ohkura S. (2012). Follicular growth and atresia in mammalian ovaries: Regulation by survival and death of granulosa cells. J. Reprod. Dev..

[37] Wojtusik J., Johnson P.A. (2012). Vitamin D regulates anti-Mullerian hormone expression in granulosa cells of the hen. Biol. Reprod..

[38] Chung M., Lee J., Terasawa T., Lau J., Trikalinos T.A. (2011). Vitamin D with or without calcium supplementation for prevention of cancer and fractures: An updated meta-analysis for the U.S. Preventive Services Task Force. Ann. Intern. Med..

[39] Manson J.E., Mayne S.T., Clinton S.K. (2011). Vitamin D and prevention of cancer—Ready for prime time?. N. Engl. J. Med..

[40] Yang Y.L., Sun L.F., Yu Y., Xiao T.X., Wang B.B., Ren P.G., Tang H.R., Zhang J.V. (2018). Deficiency of Gpr1 improves steroid hormone abnormality in hyperandrogenized mice. Reprod. Biol. Endocrinol..

[41] Burris-Hiday S.D., Scott E.E. (2021). Steroidogenic cytochrome P450 17A1 structure and function. Mol. Cell. Endocrinol..

[42] Remes T., Väisänen S.B., Mahonen A., Huuskonen J., Kröger H., Jurvelin J.S., Penttilä I.M., Rauramaa R. (2003). Aerobic exercise and bone mineral density in middle-aged finnish men: A controlled randomized trial with reference to androgen receptor, aromatase, and estrogen receptor α gene polymorphisms. Bone.

[43] Espey L.L. (1994). Current status of the hypothesis that mammalian ovulation is comparable to an inflammatory reaction. Biol. Reprod..

[44] Haussler M.R., Jurutka P.W., Mizwicki M., Norman A.W. (2011). Vitamin D receptor (VDR)-mediated actions of 1alpha,25(OH)_2_vitamin D_3_: Genomic and non-genomic mechanisms. Best Pract. Res. Clin. Endocrinol. Metab..

[45] Chen Y.Y., Powell T.L., Jansson T. (2017). 1,25-Dihydroxy vitamin D(3) stimulates system A amino acid transport in primary human trophoblast cells. Mol. Cell. Endocrinol..

[46] Zhou S., Ma Y., Zhao D., Mi Y., Zhang C. (2020). Transcriptome profiling analysis of underlying regulation of growing follicle development in the chicken. Poult. Sci..

[47] Tsutsui K., Matsunaga M., Ukena K. (2003). Biosynthesis and biological actions of neurosteroids in the Avian Brain. Avian Poult. Biol. Rev..

[48] Azziz R., Carmina E., Dewailly D., Diamanti-Kandarakis E., Escobar-Morreale H.F., Futterweit W., Janssen O.E., Legro R.S., Norman R.J., Taylor A.E. (2006). Positions statement: Criteria for defining polycystic ovary syndrome as a predominantly hyperandrogenic syndrome: An Androgen Excess Society guideline. J. Clin. Endocrinol. Metab..

[49] Zhang Y., Wang S.F., Zheng J.D., Zhao C.B., Zhang Y.N., Liu L.L., Huang J.H. (2016). Effects of testosterone on the expression levels of AMH, VEGF and HIF-1alpha in mouse granulosa cells. Exp. Ther. Med..

[50] Robinson F.E., Etches R.J. (1986). Ovarian steroidogenesis during foillicular maturation in the domestic fowl (*Gallus domesticus*). Biol. Reprod..

[51] Samardzija D., Pogrmic-Majkic K., Fa S., Glisic B., Stanic B., Andric N. (2016). Atrazine blocks ovulation via suppression of Lhr and Cyp19a1 mRNA and estradiol secretion in immature gonadotropin-treated rats. Reprod. Toxicol..

[52] Hodgins M.B., Murad S. (1986). 1,25-Dihydroxycholecalciferol Stimulates Conversion of Androstenedione into Oestrone by Human Skin Fibroblasts in Culture. J. Endocrinol..

[53] Hrabia A., Paczoska-Eliasiewicz H., Rzasa J. (2004). Effect of prolactin on estradiol and progesterone secretion by isolated chicken ovarian follicles. Folia Biol..

[54] Azhar S., Reaven E. (2002). Scavenger receptor class BI and selective cholesteryl ester uptake: Partners in the regulation of steroidogenesis. Mol. Cell. Endocrinol..

[55] Bikle D.D. (2014). Vitamin D Metabolism, Mechanism of Action, and Clinical Applications. Chem. Biol..

[56] Shahbazi M., Jeddi-Tehrani M., Zareie M., Salek-Moghaddam A., Akhondi M.M., Bahmanpoor M., Sadeghi M.R., Zarnani A.H. (2011). Expression profiling of vitamin D receptor in placenta, decidua and ovary of pregnant mice. Placenta.

[57] Heaney R.P. (2008). Vitamin D in health and disease. Clin. J. Am. Soc. Nephrol. CJASN.

[58] Jin H., Huang Y., Jin G., Xue Y., Qin X., Yao X., Yue W. (2015). The vitamin D receptor localization and mRNA expression in ram testis and epididymis. Anim. Reprod. Sci..

[59] Boisen I.M., Hansen L.B., Mortensen L.J., Lanske B., Juul A., Jensen M.B. (2017). Possible influence of vitamin D on male reproduction. J. Steroid Biochem. Mol. Biol..

[60] Johnson J.A., Grande J.P., Roche P.C., Kumar R. (1996). Immunohistochemical detection and distribution of the 1,25-dihydroxyvitamin D3 receptor in rat reproductive tissues. Histochem. Cell Biol..

[61] Gkotinakou I.M., Kechagia E., Pazaitou-Panayiotou K., Mylonis I., Liakos P., Tsakalof A. (2020). Calcitriol Suppresses HIF-1 and HIF-2 Transcriptional Activity by Reducing HIF-1/2alpha Protein Levels via a VDR-Independent Mechanism. Cells.

[62] Wu S., Xia Y., Liu X., Sun J. (2010). Vitamin D receptor deletion leads to reduced level of IkappaBalpha protein through protein translation, protein-protein interaction, and post-translational modification. Int. J. Biochem. Cell Biol..

[63] Grundmann M., Haidar M., Placzko S., Niendorf R., Darashchonak N., Hubel C.A., von Versen-Höynck F. (2012). Vitamin D improves the angiogenic properties of endothelial progenitor cells. Am. J. Physiol. Cell Physiol..

[64] Yoshizawa T., Handa Y., Uematsu Y., Takeda S., Sekine K., Yoshihara Y., Kawakami T., Arioka K., Sato H., Uchiyama Y. (1997). Mice lacking the vitamin D receptor exhibit impaired bone formation, uterine hypoplasia and growth retardation after weaning. Nat. Genet..

[65] Panda D.K., Miao D., Tremblay M.L., Sirois J., Farookhi R., Hendy G.N., Goltzman D. (2001). Targeted ablation of the 25-hydroxyvitamin D 1alpha-hydroxylase enzyme: Evidence for skeletal, reproductive, and immune dysfunction. Proc. Natl. Acad. Sci. USA.

