# Supplementary material for: 1α,25-Dihydroxyvitamin D3 Improves Follicular Development and Steroid Hormone Biosynthesis by Regulating Vitamin D Receptor in the Layers Model
Source: Curr Issues Mol Biol. 2023 May 4;45(5):4017–34. doi: 10.3390/cimb45050256 (PMC10217258; doi:10.3390/cimb45050256)
Supplement: Supplementary file 1 [file cimb-45-00256-s001.zip › cimb-2329814-SI.pdf]

Supplementary Table S1. Primers were designed according to the NCBI Gen Bank, primer sequence and PCR product length are listed.

Gene Symbol	Accession Number	Forward Sequence (5'to3')	Reverse Sequence (5'to3')	Size (bp)
CYP19	NM_001364699	TGTTCCATCACGCTATTT	GATTCTTGTTTGGGCTTC	239
CYP11A1	NM_001001756.1	GTGGACACGACTTCCATGACT	GAGAGTCTCCTTGATGGCGG	174
CYP17A1	NM_001001901	TGCTCCCTCTGCTTCAACTC	ATCCATCAGGTCCCTCACAG	255
CYP27A1	XM_040676620.1	CACCACCCTTTCAGCTCCAT	CTCCAGGTTGATGGGCTTGT	195
HSD3B1	NM_205118.1	TGGAAGAAGATGAGGCGCTG	GGAAGCTGTGTGGATGACGA	185
HSD17B1	NM_204837.1	ATGGCCTCCAAGAACACCTG	AACATCCACATGACGCTGGT	209
STAR	NM_204686.2	GTCCCTCGCAGACCAAGT	TCCCTACTGTTAGCCCTGA	196
CYP1B1	XM_015283751.3	TGCCTGTTACCATCCCACAC	GGTTGGACCATTTAGCCGGA	126
CD36	NM_001030731.1	TAACAGAAGCTGTGGGCTCC	AACAGAGCTTCCTGGCACAA	175
VLDLR	NM_205229.1	AGACTGTTCAGACGGCAGTG	CACTGGGTTGACTGAGGACC	213
ABCA1	NM_204145.2	CGCCGTTTCTTGTGGAACTG	CAGACACCTGAATCGCCCAT	138
SPP1	NM_204535.4	ATTTGACAGCCCTGAGGTGG	CAATGCTGTGGCGATCTTCG	146
PLTP	NM_001162406.1	GAAGTATGCGTGCATGGCTG	CAGCAAGAGCAAGCCAACAG	155
WNT6	NM_001007594.2	GACAGAGTGCAAATGCCACG	GAGATCCTGCTTGTCGGGAG	196
NR5A1	NM_205077.1	GAGGATCTGGACGAGCTGTG	TCTTGCAGTTCTGGCTCTCG	151
BMP15	NM_001006589.2	GACAAAAGCGACTGCTCCCT	CTTACAGTAGCGCGGGTTGT	105
PTGS2	NM_001167719.1	AGGAGCATCCAGAGTGGGAT	TTCAGCTGCGATTCGGTTCT	188
LHX9	NM_205426.1	ATCCCAGAAGACAAAGCGCA	TTGGCATCTGGGTTGTGGTT	96
ZP4	NM_204879.1	GGAGCTTGCAGCTCACCTTA	CGGACAGCAGAACAGACACT	331
CEL	NM_001012997.1	CCAAACTGATGTCCGTGGGA	CCGTAGTTCCCTGGCATGTT	279
VDR	NM_205098	AGTCAGCGATGTCACCCAAG	TCTGGAGGATGTCCGAGAGG	206
FSHR	NM_205079	GAGCGAGGTCTACATACA	GCACAAGCCATAGTCA	281
ESR1	NM_205183.2	GCCTGGCAGGATTTCACTCT	GCCTCCCTCATCCCAAAGCT	154
ESR2	NM_204794.2	TGTGGGTATCGAATCCTGCG	ACATTGGGAGGCTCAGCTTC	161
CDK2	NM_001199857	ACGTGATCCACACGGAGAAC	GCAGCTGGAACAGGTAGCTC	132
CCNE1	NM_001031358	AGGTTTATGGCAACACAACAGAA	AACTGGTGCAACTTTGGTGG	116
CCND1	NM_205381	ATAGTCGCCACTTGGATGCT	AACCGGCTTTTCTTGAGGGG	122
CDKN1A	NM_204396	TACGAAGCAATGCCGAGTCT	TCAGTCCCTTCCGTGGTCTT	116
CDKN1B	NM_204256	GAGCCCGAGACGACATCAAA	TCCCATGGAGACCGACGATA	133
ATM	NM_001162400	TCCCGCATTTTCTCGCAGAT	TCAAGGGAAGAGGCGTTGAC	123
BCL2	NM_205339.3	GCTGCTTTACTCTTGGGGGT	CTTCAGCACTATCTCGCGGT	128
CASP3	NM_204725.2	CAGCTGAAGGCTCCTGGTTT	GCCACTCTGCGATTTACACG	106
CASP9	XM_424580.7	AGCTGTTGACAAGAGTGACCA	TTCCGCAGCTCCACATCAAT	223
GAPDH	NM_204305	GAGGGTAGTGAAGGCTGCTG	CACAACACGGTTGCTGTATC	199

Supplementary Table S2. Different expressed genes are involved in ovarian steroidogenesis and cholesterol metabolism of chicken SYFs.

Gene ID	Gene symbol	CON-LVD		CON-HVD		LVD-HVD	
		log2fc	*P* value	log2fc	*P* value	log2fc	*P* value
ncbi_414854	CYP19A1	0.686	0.014	-0.023	0.942	-0.709	0.252
ncbi_425056	CYP17A1	0.069	0.539	0.109	0.446	0.041	0.898
ncbi_431683	CYP27A1	0.034	0.579	0.346	0.028	0.313	0.144
ncbi_396015	HSD3B1	0.242	0.231	0.025	0.800	-0.217	0.381
ncbi_417165	HSD17B1	0.527	0.120	-0.257	0.543	-0.784	0.031
ncbi_395421	STAR	0.089	0.792	0.833	0.204	0.743	0.199
ncbi_421466	CYP1B1	0.276	0.116	0.189	0.332	-0.087	0.493
ncbi_417730	CD36	0.393	0.573	0.921	0.028	0.528	0.552
ncbi_396154	VLDLR	0.056	0.406	0.351	0.018	0.295	0.089
ncbi_373945	ABCA1	0.354	0.256	0.749	0.008	0.395	0.343
ncbi_423176	ASTL	-0.073	0.986	1.221	0.075	1.294	0.092
ncbi_428164	PLTP	0.391	0.060	0.642	0.015	0.251	0.468
ncbi_395235	WNT6	0.305	0.246	0.659	0.034	0.354	0.376
ncbi_395960	NR5A1	0.259	0.129	0.922	0.080	0.663	0.088
ncbi_428697	BMP15	-1.573	0.0004	-0.539	0.345	1.034	0.078
ncbi_396451	PTGS2	-0.832	0.005	-0.219	0.650	0.613	0.135
ncbi_396397	LHX9	-0.241	0.845	-0.818	0.018	-0.577	0.219
ncbi_395692	ZP4	-1.752	7.17E-05	-1.038	0.025	0.713	0.253
ncbi_417165	CEL	-0.412	0.565	1.450	0.004	1.862	0.0001
ncbi_418307	TRPV6	0.696	0.184	1.028	0.029	0.333	0.580

Supplementary Figure S1

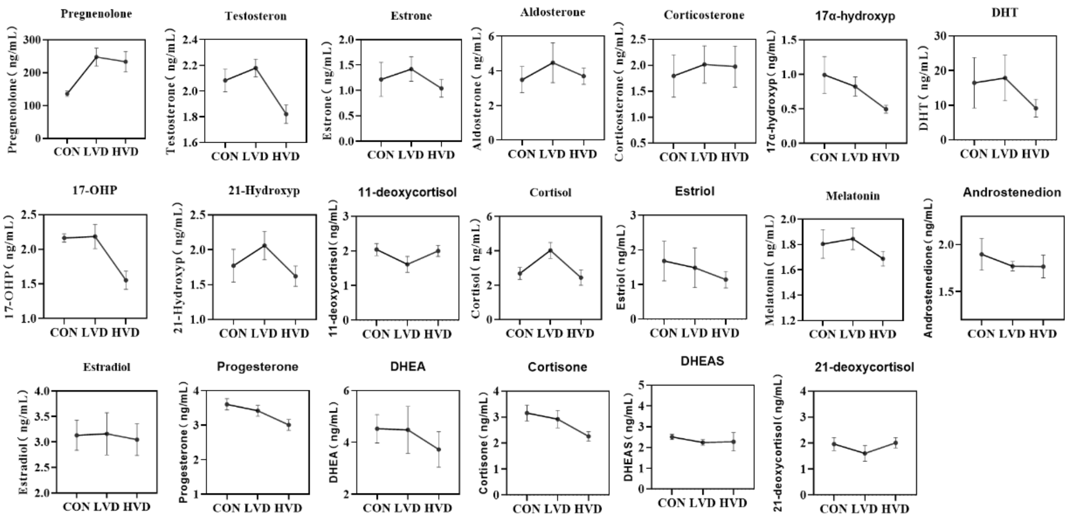

Supplementary Figure S1. The concentration distribution of differential 20 steroid hormones in layers SYF fluid among the three groups. Values expressed as means ± SEM (n=6). CON: with calcitriol 0 μg/kg BW; LVD: with calcitriol 10 μg/kg BW; HVD: with calcitriol 100 μg/kg BW; VitD3: 1α, 25-dihydroxy vitaminD3.

Supplementary Figure S2

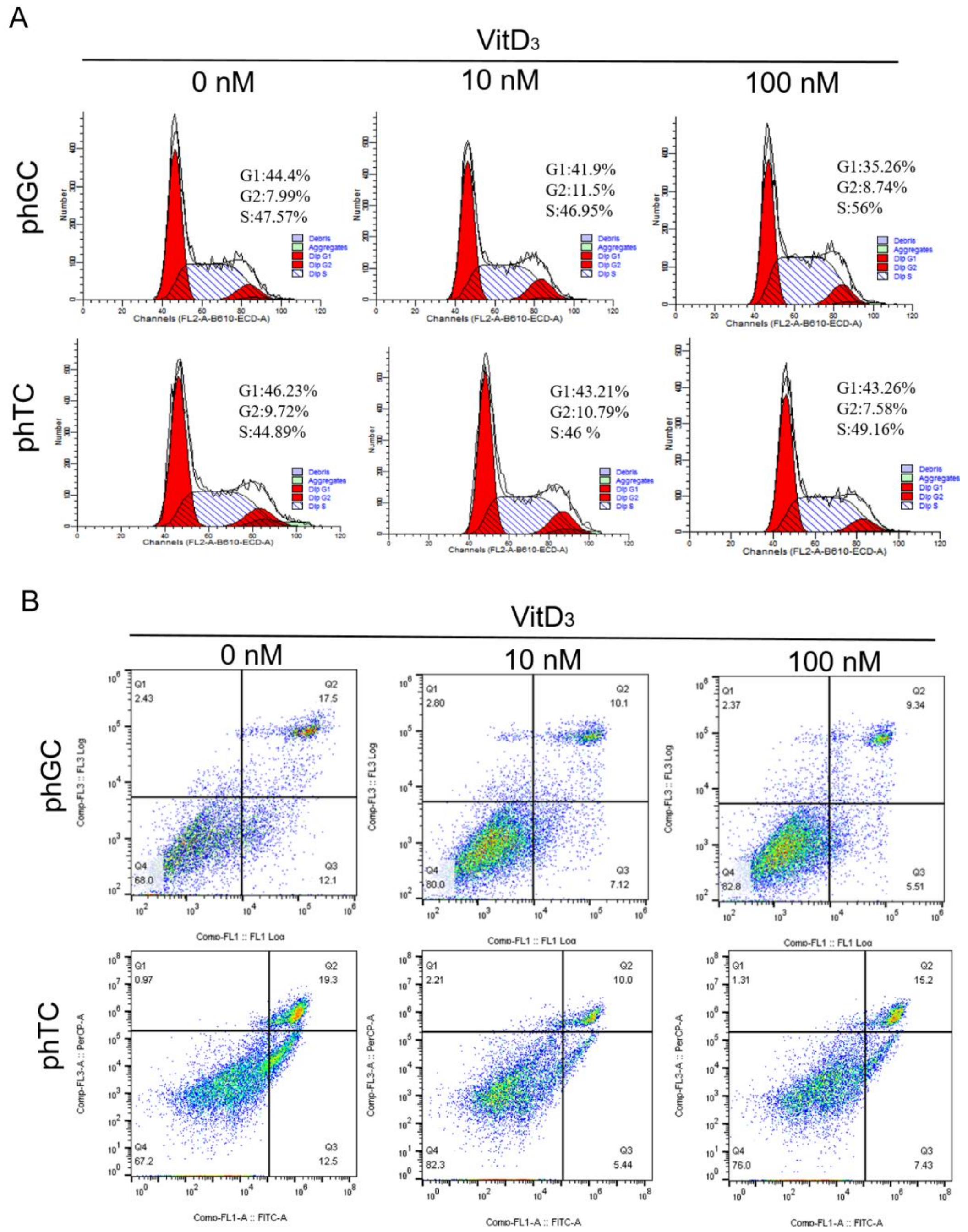

Supplementary Figure S2. Flow cytometric analysis of cell cell and cell apoptosis in phGCs and phTCs among the three groups. (A) Flow cytometric analysis of cell cycle in phGCs and phTCs after VitD3 treatment. The percentages of cells in the G1, G2, and S phases were determined of cell cycle progression in phGCs and phTCs. (B) Flow cytometric analysis of cell apoptosis in phGCs and phTCs after VitD$_3$ treatment. Flow cytometry was utilized to analyze cell apoptosis, employing AnnexinV+/PI method. The percentages of apoptosis (AnnexinV+/PI−and AnnexinV+/PI+) cells data were presented as the means ± SEM (n=3). Multiple comparisons were carried out using Duncan's post hoc comparison. Cultured phGCs and phTCs were treated with VitD3 at 0, 10, and 100 nM for 24 h. phGCs: granulosa cells from

prehierarchical follicles; phTCs: theca cells from prehierarchical follicles; $VitD_3$: 1α, 25-dihydroxy vitaminD_3.
